# Dr. William Henderson Wilkes

**Published:** 1896-09

**Authors:** 


### DOCTOR WILLIAM HENDERSON WILKES.

The profession of Texas has sustained a sad loss in the death of this eminent and greatly beloved physician. He died at his home in Waco August 14, 1896.

Dr. Wilkes was a general favorite with the doctors, and equally popular with all classes wherever he was known. Genial, kind-hearted, witty, and of a decidedly social turn, he could not have been other than an agreeable and welcome companion. Able and gifted as a physician, he naturally took high rank in his profession, and did a large practice. Socially his good qualities were greatly enjoyed, for he was ever ready with a witty or spirited reply, and on convivial occasions, such as at banquets, often enjoyed by the doctors at their meetings, he was in great demand—was always ready with a speech. He was a *leader*, socially as well as in his profession. He took an active interest in matters concerning his city and county, and was twice elected mayor of Waco. An evidence of his versatility and popularity is found in the fact that he filled the office of mayor twice, and, so far as is known, never made an enemy. Much of the growth of the "Artesian City" is attributed to his wise management of the city's affairs.

But as large as was his practice, and as active as was his life, Dr. Wilkes died a comparatively poor man. He was one of the few of the survivors of the old *rigime* who considered the profession of medicine a mission, and not a trade. His fees were

"honoraria," and not "doctor bills." He made no effort to collect for his services, but took what was given him, and if the patient did not ask for his bill, he had none. Of course he kept account of his services, and valued them as others do, but he was never known to make any effort to force a collection. As the tender and sympathetic physician, the wise friend and counselor, the social companion, and the conservative business man, Dr. Wilkes will be missed and mourned by all classes. He was a man of great and varied talent. A profound student of medicine and a ready speaker, his services were in request as a lecturer. He was tendered and accepted the chair of obstetrics and diseases of women in the Kansas City Medical College, and for a short while (in 1888) occupied it, but preferring private life, as well as for other reasons, he resigned, and returned to Waco. In 1889 he was tendered but declined the chair of practice in the medical department of the University ot Texas (Galveston). In 1891 Dr. Wilkes was elected president of the Texas State Medical Association. His address on the occasion of the succeeding meeting will be found in the Transactions of that body (1888).

Dr. Wilkes had quite a creditable record also as a soldier. He was born in Raymond, Hinds county, Miss., in 1833 (April 8), and was, at his death, in his sixty-third year.

He was the only son of Richard S. Wilkes, of Marshall county, Tenn., and was a descendant of the "Virginia emigrant," John Wilkes. Upon the death of his father (in 1834), when the son was only one year old, his mother, whose maiden name was Nancy C. Henderson, of the North Carolina Hendersons, removed to Tennessee and settled in Giles county, the former home of the father. Here young Wilkes was reared, and until 15 years of age worked upon a farm. Later he studied medicine with Drs. Kennedy & Pugh, at Cornersville, Tenn., and graduated from the medical department of the University of Nashville in 1855. Settling in Marshall county, Tenn., he was engaged in practice when the war came on. Dr. Wilkes volunteered as a private in the Confederate army December 1, 1861, 23rd Tennessee Regiment, and upon organization of Company F, he was elected captain. He was captured with the entire regiment at Fort Donaldson, and for seven months was a prisoner at Indianapolis. On being exchanged at Vicksburg, and reorganization of the command at Jackson, Miss., October, 1862, Dr. Wilkes was elected colonel of the regiment. This position he was finally compelled to resign in consequence of bad health, the result of his long imprisonment, having suffered a wound through the shoulder by a minnie ball during the engagement at Fort Donaldson. He served in Mississippi and Tennessee and Louisiana under Hindman, Maxey, Quarles and Gardner, and participated in the battles of Fort Donaldson, Port Hudson and other engagements. During several engagements Colonel Wilkes commanded the brigade to which his regiment was attached.

He removed to Waco in 1868, where he at once became one of the leading practitioners. He owned an interest in the *Morning Examiner*, and amongst other services edited the paper a part of the time. This further shows the versatility of his talent. He became a Mason in 1854, and had taken all the Chapter, Council and Commandery degrees. He served as worshipful master in Tennessee and Texas.

Dr. Wilkes also took an active interest in politics, and was chairman of the democratic executive committee of McLennan county, and chairman of the delegation to the State convention from that county in 1888.

Dr. Wilkes had been twice married. His first wife, to whom he was marridd in Cornersville, Tenn., in 1855, was Miss M. A. Holt, of Marshall county, Tenn. This estimable lady died in Waco, December 21, 1883, when Dr. Wilkes was serving his first term as mayor of that city. Six children were born of this union, three of whom died in infancy, and three survive their father—Mrs. W. R. Shaw, John R. Wilkes and Dr. W. O. Wilkes, of Waco. In April, 1887, he was again married, his second wife being Miss Irene McConnell, of Liberty, Tenn. Mrs. Wilkes, on account of whose health, partly, it was that Dr. Wilkes resigned the chair of obstetrics and diseases of women in the Kansas City Medical College and returned to Waco, survives him, and resides in that city. To her and the others of the bereaved family, the sympathy of the JOURNAL goes out, as well as that of the entire medical profession of the State, who held the doctor in the highest regard. His loss is a serious bereavement of the profession, and he will be missed at our annual reunions, where he was most always the "life of the occasion."

In religion Dr. Wilkes was a Methodist. He was a member of the Masonic Fraternity, as stated, and of the Knights Templar. He was also a member of the Confederate Veterans, of Pat Cleburn camp.

The following resolutions were adopted by the Waco Medical Association, and the Association held a memorial service the week following the funeral:

WHEREAS, In the dispensation of Divine Providence our honored and esteemed fellow-member of the Waco Medical Association, Dr. W. H. Wilkes, has been called from earthly labor to a well earned heavenly rest; therefore, be it resolved by the Waco Medical Association:

1. In the death of Dr. Wilkes our Association has lost one of its most honored and influential fellows, the profession an able and conscientious physician, society an upright and cultured man, the State a patriotic and useful citizen.

2. We deplore the loss of this Christian citizen and physician, and extend to his bereaved family the sincere condolence and sympathy of the Waco Medical Association.

3. That this Association instruct the secretary to spread these resolutions on the minutes, and a copy be furnished the family of our deceased brother, and also a copy be furnished the public press.

J. C. KING, Chairman,
H. C. BLACK,
W. A. HOWARD,
M. L. GRAVES.

